# Integrated targeted metabolomic and lipidomic analysis: A novel approach to classifying early cystic precursors to invasive pancreatic cancer

**DOI:** 10.1038/s41598-019-46634-6

**Published:** 2019-07-15

**Authors:** Rogier Aäron Gaiser, Alberto Pessia, Zeeshan Ateeb, Haleh Davanian, Carlos Fernández Moro, Hassan Alkharaan, Katie Healy, Sam Ghazi, Urban Arnelo, Roberto Valente, Vidya Velagapudi, Margaret Sällberg Chen, Marco Del Chiaro

**Affiliations:** 1 Division of Clinical Diagnostics and Surgery, DENTMED, Karolinska Institutet, Huddinge, Sweden; 2 Metabolomics Unit, Institute for Molecular Medicine Finland (FIMM), HiLIFE, University of Helsinki, Helsinki, Finland; 3 Division of Surgery, CLINTEC, Karolinska University Hospital, Stockholm, Sweden; 4 Department of Clinical Pathology/Cytology, Division of Pathology, Karolinska University Hospital, Huddinge, Sweden; 5 Division of Pathology, LABMED, Karolinska Institutet, Huddinge, Sweden; 6 Department for Digestive Diseases, Sapienza University of Rome, Rome, Italy; 7 Division of Surgical Oncology, Department of Surgery, University of Colorado Denver, Aurora, CO, USA; 8 Tenth People’s Hospital, Tongji University, Shanghai, China; 9 College of Dentistry, Prince Sattam bin Abdulaziz University, Al-Kharj, Saudi Arabia

**Keywords:** Lipidomics, Metabolomics, Diagnostic markers, Tumour biomarkers, Pancreatic cancer

## Abstract

Pancreatic cystic neoplasms (PCNs) are a highly prevalent disease of the pancreas. Among PCNs, Intraductal Papillary Mucinous Neoplasms (IPMNs) are common lesions that may progress from low-grade dysplasia (LGD) through high-grade dysplasia (HGD) to invasive cancer. Accurate discrimination of IPMN-associated neoplastic grade is an unmet clinical need. Targeted (semi)quantitative analysis of 100 metabolites and >1000 lipid species were performed on peri-operative pancreatic cyst fluid and pre-operative plasma from IPMN and serous cystic neoplasm (SCN) patients in a pancreas resection cohort (n = 35). Profiles were correlated against histological diagnosis and clinical parameters after correction for confounding factors. Integrated data modeling was used for group classification and selection of the best explanatory molecules. Over 1000 different compounds were identified in plasma and cyst fluid. IPMN profiles showed significant lipid pathway alterations compared to SCN. Integrated data modeling discriminated between IPMN and SCN with 100% accuracy and distinguished IPMN LGD or IPMN HGD and invasive cancer with up to 90.06% accuracy. Free fatty acids, ceramides, and triacylglycerol classes in plasma correlated with circulating levels of CA19-9, albumin and bilirubin. Integrated metabolomic and lipidomic analysis of plasma or cyst fluid can improve discrimination of IPMN from SCN and within PMNs predict the grade of dysplasia.

## Introduction

Pancreas cancer (PC) is expected to become the second most common cause of cancer related death within the next decade^1^. Contrary to other cancer types such as breast^2^ and colorectal^3^, whose prognosis has been progressively improving over the time, pancreatic cancer prognosis remains poor. This has in part been due to the lack of an effective screening method with the ability to identify pancreatic lesions that are at risk of progression and that appear before PC develops^4^.

Pancreatic cystic neoplasms (PCNs) are increasingly diagnosed and display a prevalence as high as 45% in the general population^5^. Intraductal Papillary Mucinous Neoplasm (IPMN) account for half of all PCNs and are increasingly considered possible precursor lesions of PC^6–8^. IPMNs can progress from low-grade dysplasia (LGD) through high-grade dysplasia (HGD) to invasive cancer^9,10^. However, as the prevalence of IPMNs in the general population is higher than the incidence of PC, only a minority of patients affected by IPMNs will develop PC^6,8,10^. Therefore, the detection of IPMNs by currently available imaging techniques is an opportunity for early diagnosis of neoplastic precursor lesions and prevention of PC. However, the development of a population-based screening program is challenged by two factors: on one hand the costs of lifelong surveillance, on the other hand the low pre-operative diagnostic accuracy for pancreatic cystic lesions. These two problems partly overlap, as due to the low diagnostic yield of conventional radiology, many patients will undergo unnecessary lifelong follow-up with magnetic resonance imaging and/or endoscopic ultrasound with associated high health care costs that might become particularly unsustainable in the near future^11^.

Current indications for surgery in IPMN patients are mainly based on the pre-operative radiological imaging that suffers from low accuracy (60–70%)^12^. Diagnostic yield can be slightly increased by adding endoscopic ultrasound-guided fine-needle aspiration (EUS-FNA), which allows for cytological and carcinoembryonic antigen (CEA) analyses^13^. Nonetheless, fluid cytology does not allow differentiation between different types of mucinous cysts or between different grades of dysplasia in IPMN, and CEA is inaccurate to discriminate benign mucinous cysts and cysts with high-grade dysplasia or an associated invasive carcinoma^14–16^. Thus, the pre-operative diagnostic accuracy to distinguish between the various benign or (pre-)malignant PCNs, such as IPMNs, is low, and there are no methods available to discriminate between the different grades of dysplasia associated with IPMNs. Correctly identifying PCNs and their risk for progression to cancer is clinically crucial; as such, novel biomarkers from blood or cyst fluid may allow for a more accurate definition of IPMNs and improve their management and treatment^17–20^.

Metabolic reprogramming is an established hallmark of cancer^21^. In addition to the carbohydrate and amino acid nutrients required by growing cancer cells, the lipid-scavenger pathway and *de novo* fatty acid synthesis are important for maintaining cancer cell proliferation and survival in the tumor environment^22,23^. Development and progression of PC is associated with alterations in circulating metabolic profiles^24–29^. Whilst previous studies have compared the metabolic profiles of PC patients and healthy individuals^25,26,28^, few have examined IPMN patients or considered the spectrum of IPMN severity, which is relevant for pancreatic surgery management^27,30^.

This study aimed at defining the metabolomic and lipidomic makeup of pancreatic cyst fluid and plasma in pancreas resection patients with IPMN and serous cystic neoplasm (SCN).

## Results

### Study characteristics

This cohort study included 35 patients undergoing pancreas resection, from whom pre-operative blood plasma (n = 21) and peri-operative cyst fluid (n = 31) were collected (Supplementary Fig. S1). Following histological validation of resected tissues, four groups were assigned: serous cystic neoplasm (SCN), IPMN with low-grade dysplasia (LGD), IPMN with high-grade dysplasia (HGD), and invasive IPMN (Cancer) for which clinical parameters are summarized in Table 1. As expected, the IPMN group was older, of mixed gender, and had comparable BMI with SCN controls. Cardiovascular disease (CVD) and diabetes were more common in patients with IPMN. Compared to SCN, IPMN LGD and HGD showed no significant elevation of circulating CA19-9, HbA1c, amylase, albumin, bilirubin, or white blood cell count. Only Cancer had significantly increased circulating CA19-9 or HbA1c levels.

**Table 1:** Patient group characteristics.

	Cyst fluid (n = 31)				Plasma (n = 21)			
	SCN	IPMN			SCN	IPMN		
		LGD	HGD	Cancer		LGD	HGD	Cancer
Patients, % (n)	16.1 (5)	25.8 (8)	22.6 (7)	35.5 (11)	23.8 (5)	23.8 (5)	28.6 (6)	23.8 (5)
Female, %	100	50*	42.9*	27.3**	100	20*	33.3*	40
Alcohol use, %	60	25	28.6	18.2	40	20	16. 7	60
Smokers, %	40	0	0	9.1	40	0	0	40
CVD, %	20	71.4	71.4	54.6	20	60	50	60
Statins use, %	20	12.5	14.3	9.09	20	0	16.7	40
Diabetes, %	0	12.5	42.9	36.4	0	20	16.7	20
Age, years	48	66**	72***	69***	53	65	72.5**	69**
median (range)	(34–58)	(56–81)	(66–75)	(46–83)	(34–68)	(56–76)	(66–75)	(65–83)
BMI, kg/m^2^	29.64	27.51	27.21	24.97	28.01	32.16	24	25.69
median (range)	(24.1–32.0)	(21.8–36.6)	(23.4–28.3)	(20.2–29.7)	(24.1–31.0)	(24.8–36.6)	(21.5–28.3)	(24.1–32.9)
HbA1c, mmol/mol	31	42.5	38	43*	33	44	38	51.5
median (range)	(30–37)	(35–48)	(31–55)	(31–67)	(30–43)	(37–48)	(31–55)	(31–81)
S-CA 19–9, kE/L	11	18	11	376*	11	11	16	285**
median (range)	(6.8–62)	(6.4–182)	(<1–115)	(<1–1040)	(7.9–62)	(6.4–182)	(<1–115)	(46–480)
Serum amylase, µkat/L	0.3	0.41	0.24	0.25	0.31	0.44	0.195	0.27
median (range)	(0.19–1.64)	(<0.13–0.65)	(<0.13–0.93)	(<0.13–0.87)	(0.19–1.64)	(<0.13–0.54)	(<0.13–0.72)	(<0.13–0.54)
Albumin, g/L	36	36	36	31	38	37	34.5	31.5
median (range)	(33–39)	(26–38)	(22–39)	(19–38)	(33–39)	(36–39)	(22–39)	(28–34)
Bilirubin, µmol/L	6	6.5	5	24	6	8	8	30
median (range)	(3–18)	(<3–13)	(<3–315)	(5–150)	(3–7)	(4–13)	(4–315)	(12–119)
WBC, × 10^9^/L	6.3	7.45	7.8	9.8	6.3	7.5	8.3	11.2**
median (range)	(4.4–9.2)	(5–9.4)	(5.6–12.9)	(5–13.9)	(4.4–9.2)	(5.3–9.4)	(7.2–12.9)	(8–13.9)

Statistical comparisons between each group and the control group (SCN) were made using one-way ANOVA with Dunnett’s multiple comparisons test for quantitative parameters and chi-square test for qualitative values; *p ≤ 0.05, **p ≤ 0.01, ***p ≤ 0.001.

### Metabolite profiling reveals alterations of lipid metabolism pathways

**Figure 1:**
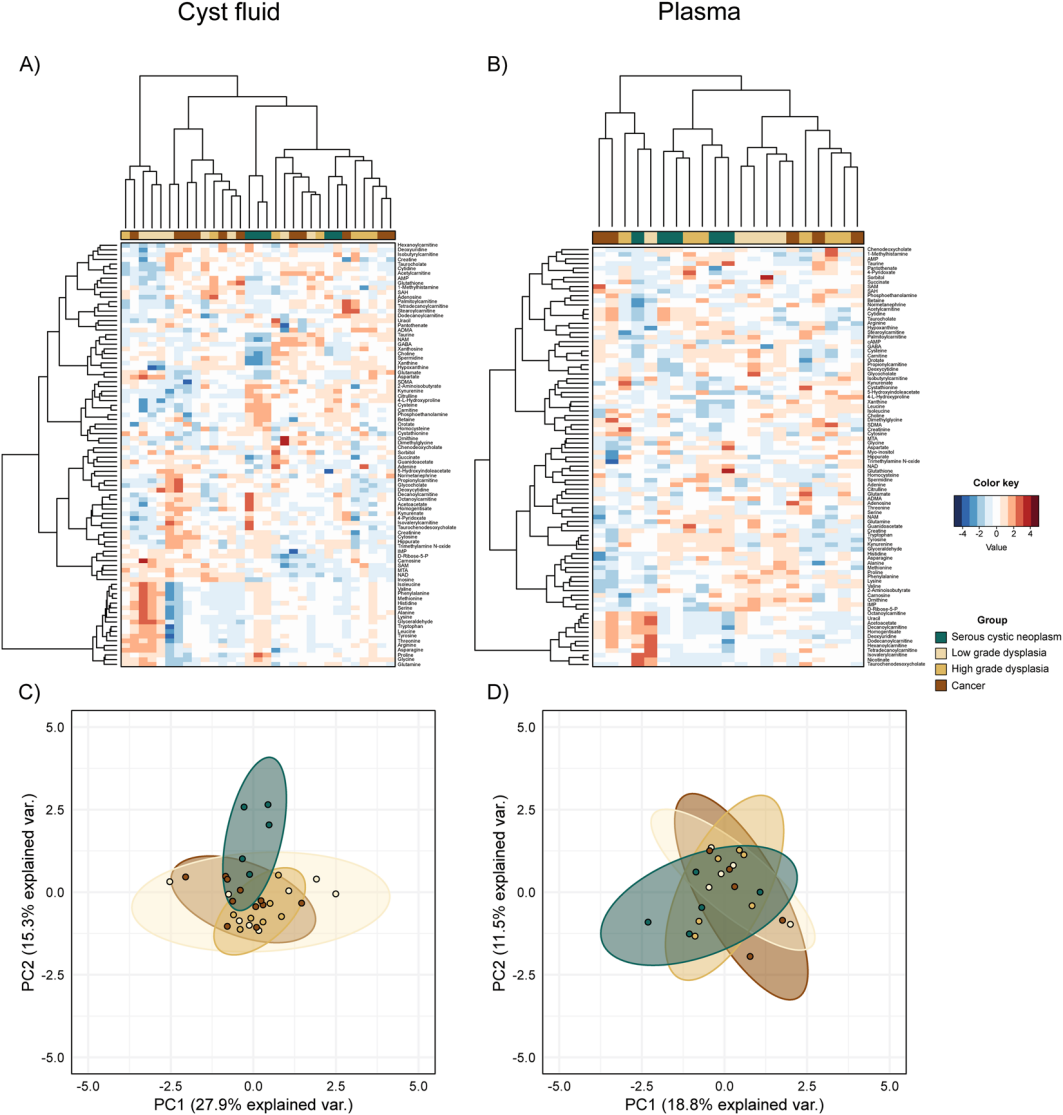
Metabolomic profile. Heatmap of cyst fluid **(A)** and plasma **(B)** metabolite concentrations. Projection of patient samples on the first two principal components (PCA) for cyst fluid **(C)** and plasma **(D)** datasets. Data (cyst, n = 31; plasma, n = 21) were adjusted for confounding factors and features were subsequently standardized to have a mean of zero and unit variance. Dendrograms were built using the Euclidean distance matrix and Ward’s method.

Cyst fluid and plasma were profiled single-blinded using a targeted and (semi)quantitative liquid chromatography-tandem mass spectrometry (LC-MS/MS) method. A total of 90 and 91 different metabolites were measured in cyst fluid and plasma, respectively. A hierarchical clustered heatmap of the metabolomic data showed no clear grouping of metabolite profiles according to diagnose group (Fig. 1A,B). However, principal component analysis (PCA) showed that cyst fluid but not plasma from SCN was dissimilar to all other groups (Fig. 1C,D).

We next applied quantitative metabolic pathway enrichment analysis, using metabolite identities (see Supplementary Table S1). Because HGD and Cancer are target groups for resection, these groups were combined (HGD/Cancer). Compared to SCN, 34 enriched pathways were found in cyst fluid from HGD/Cancer and 12 enriched pathways from LGD at a significance level of 0·05 (Supplementary Fig. S2). Among these, lipid pathways appeared to dominate, e.g. phosphatidylethanolamine biosynthesis, phosphatidylcholine biosynthesis, taurine and hypotaurine metabolism, phospholipid biosynthesis, beta oxidation of very long chain fatty acids, fatty acid metabolism, oxidation of branched chain fatty acids, and sphingolipid metabolism. Several of these were also significantly enriched in plasma samples of HGD/Cancer or LGD compared to SCN, including sphingolipid metabolism, phosphatidylethanolamine biosynthesis, phosphatidylcholine biosynthesis (Supplementary Fig. S2).

### Lipidomic profiling indicates a difference between IPMNs and SCN

**Figure 2:**
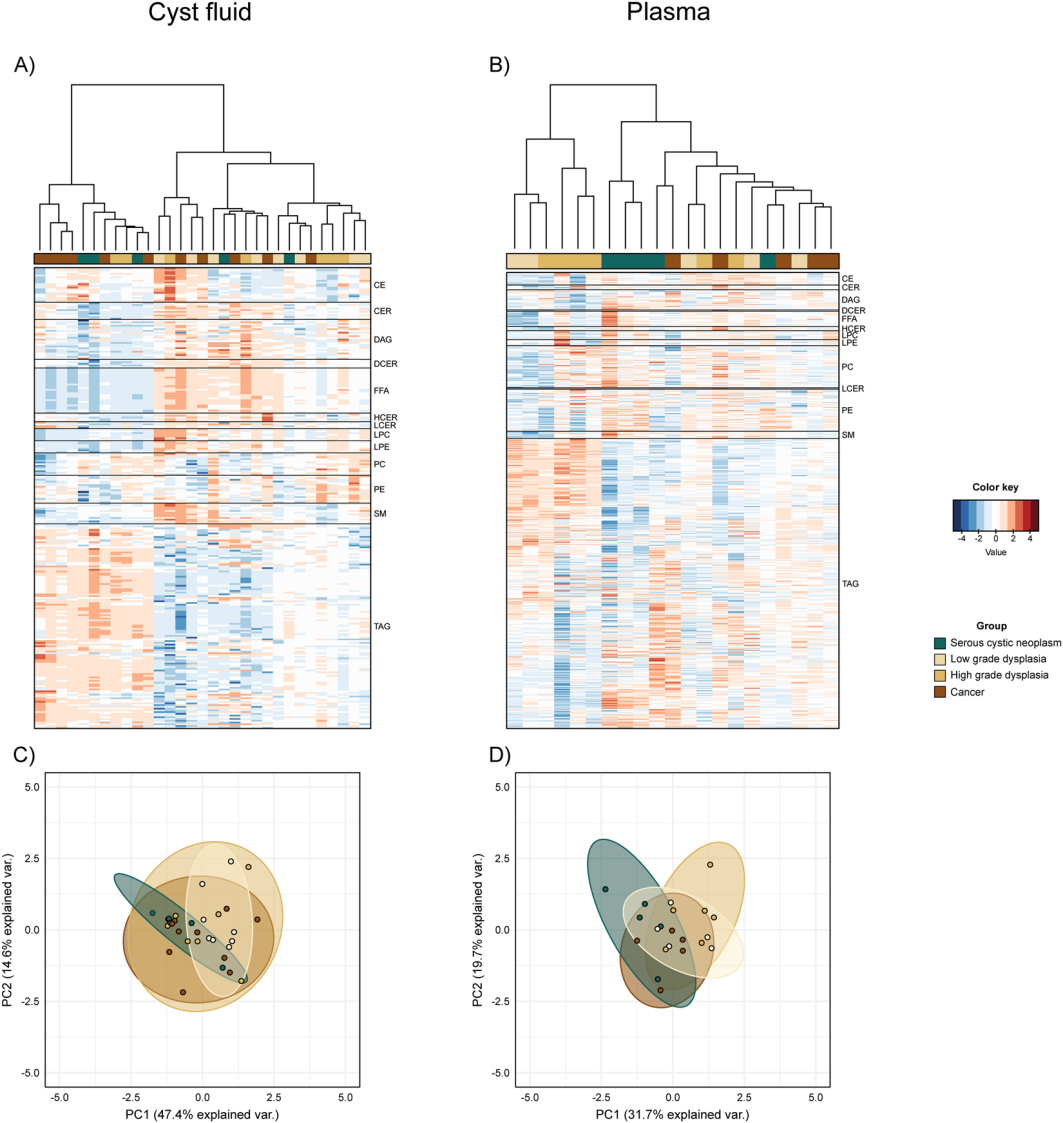
Lipidomic profile. Heatmap of cyst fluid **(A)** and plasma **(B)** lipid concentrations. Projection of patient samples on the first two principal components (PCA) for cyst fluid **(C)** and plasma **(D)** datasets. Data (cyst, n = 31; plasma, n = 21) were adjusted for confounding factors and features were subsequently standardized to have a mean of zero and unit variance. Dendrograms were built using the Euclidean distance matrix and Ward’s method.

**Figure 3:**
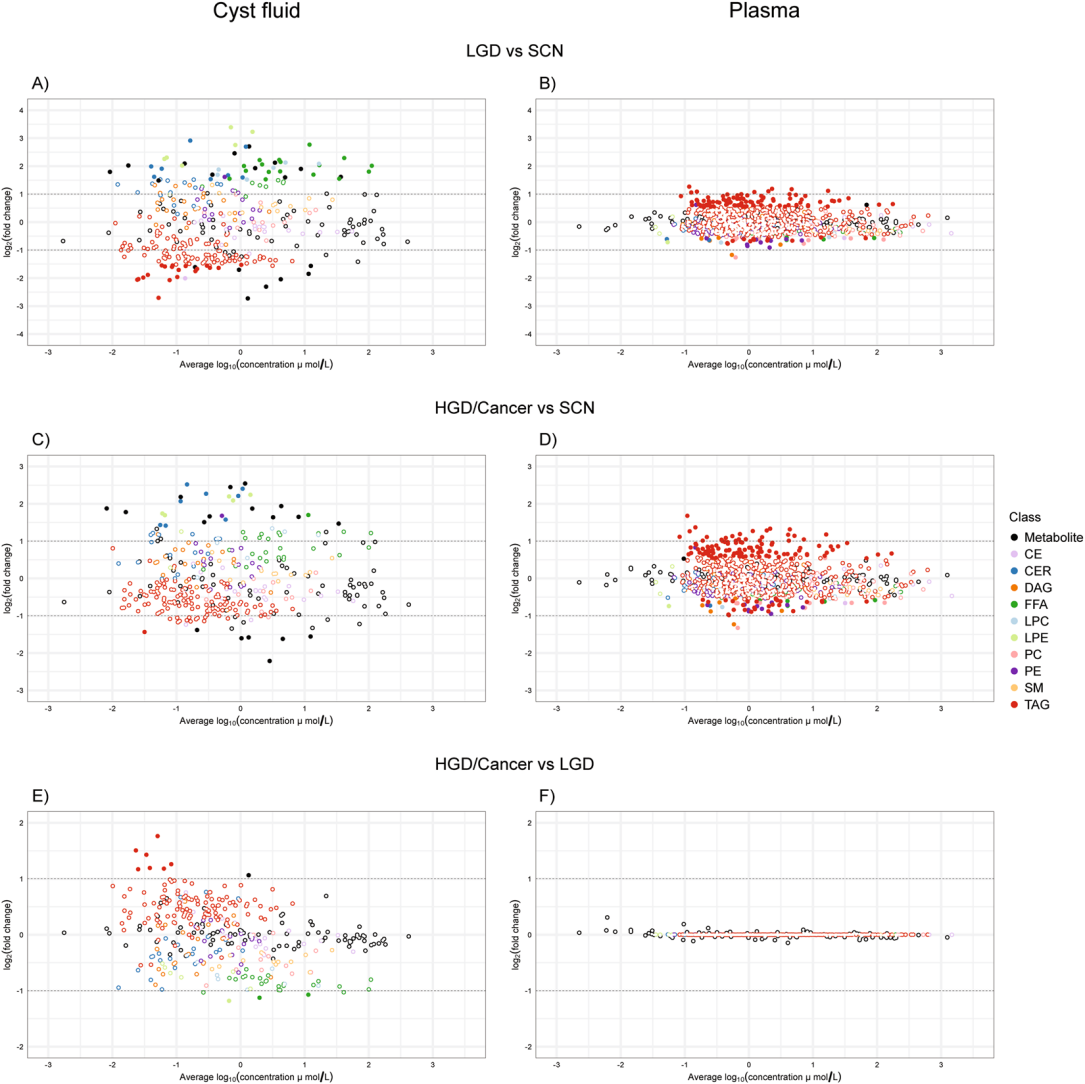
Estimated fold changes of concentrations of all measured analytes (including metabolite and lipid molecular species) between selected groups. LGD compared to SCN in cyst fluid **(A)** and plasma **(B)**. HGD/Cancer compared to SCN in cyst fluid **(C)** and plasma **(D)**. HGD/Cancer compared to LGD in cyst fluid **(E)** and plasma **(F)**. Filled dots are fold changes whose credibility interval does not overlap with the null reference value of one-fold change, or zero on the plotted log scale.

As metabolic analysis of IPMN indicated altered lipid metabolism, and considering the pancreatic exocrine function of secreting lipases that might be affected in PCN patients, we next performed a high definition lipid profiling of paired aliquots using the SCIEX Lipidyzer™ technology, where 1100 lipid molecular species were measured in all the samples. Out of those 1100 measured lipid molecular species, we successfully detected and quantified a total of 430 in cyst fluid and 941 in plasma. Heatmap visualization showed that triacylglycerol (TAG)-related lipids are the most abundant type among all lipid classes in both cyst fluid and plasma (Fig. 2A,B). Lipidomic profiles of HGD and Cancer appear to be similar to each other, while LGD was clustered slightly differently. Although the lipidomic profiles of plasma and cyst fluid of the SCN group, as projected on the first two principal components (PCA), show a different direction and shape of the clouds of points when compared to IPMN groups, no evidently clear separation of diagnose groups can be observed from the 2D plot (Fig. 2C,D). The lipidomic analyses suggest alterations in lipid compound composition in cyst fluid and plasma, pointing to the possibility of discrimination of pancreatic disease severity, confirming the pathway enrichments we observed. Looking at fold change estimations (Fig. 3 and Supplementary Table S1) it is possible to observe a clear alteration of the TAG class in plasma samples in both LGD and HGD/Cancer compared to SCN. In IPMN cyst fluid samples, instead, free fatty acids (FFA) and ceramides (CER) appear to have, on average, higher concentrations than those of SCN samples while having lower amount of TAGs. Interestingly, the profile of the Cancer group is similar to that of LGD in plasma and to that of HGD in cyst fluid, while only TAGs differ significantly between HGD and LGD in both plasma and cyst fluid (Supplementary Fig. S3).

### Integrated metabolite and lipid data predict IPMN disease groups

**Figure 4:**
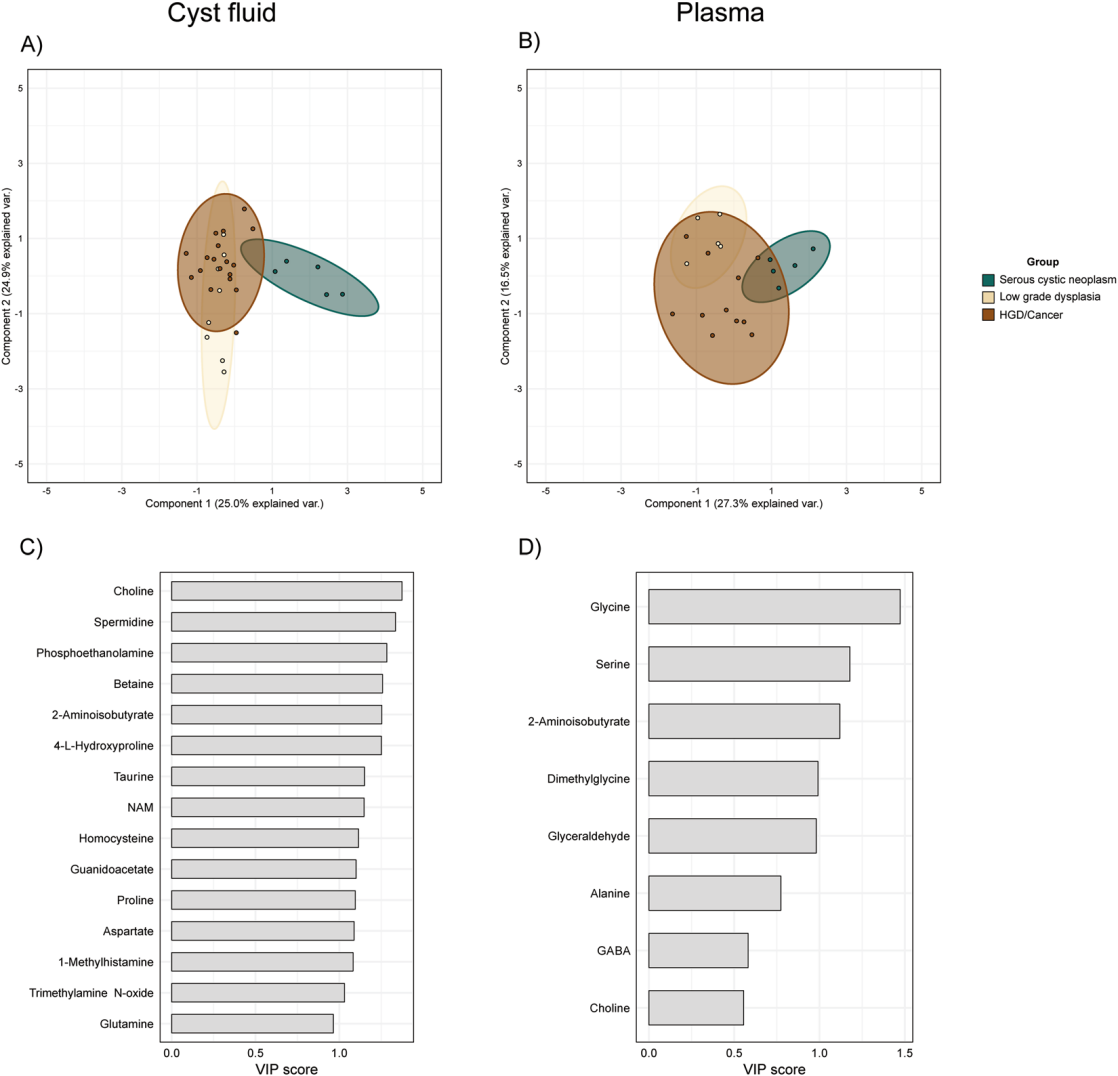
Canonical Powered Partial Least Squares and Discriminant Analysis (CPPLS-DA) results. Projection of patient samples on the first two principal components in cyst fluid **(A)** and plasma **(B)**. Highest variable importance in projection (VIP) scores in cyst fluid **(C)** and plasma **(D)**.

Accurate classification of IPMN severity using novel biomarkers in cyst fluid or plasma may facilitate the discrimination of low-risk from high-risk patients. We therefore assessed the predictive capacity of the integrated metabolome and lipidome profiles to classify samples according to their corresponding disease group. As IPMN HGD and Cancer are considered as high-risk lesions, we combined these into a single group. Effects of clinical covariates (Table 1) were estimated and subtracted from the raw data prior to analysis, and the only covariates that improved the classification model were age and BMI. The result of binary classifications and the performance of the CPPLS-DA model are given in Table 2 and Supplementary Table S2. The model discriminated between SCN and IPMN with very high accuracy (100%) in both cyst fluid and plasma samples. Choline, 2-aminoisobutyrate, trimethylamine n-oxide, glycine, alanine, and glyceraldehyde were found to be essential discriminatory molecules in both cyst fluid and plasma. Furthermore, dimethylglycine was a discriminatory compound for cyst fluid while serine and GABA were for plasma. Overall, the two biofluids displayed similar predicting power, with cyst fluid-based classification performing slightly better (accuracy of approximately 90%) when classifying the three groups SCN, LGD and HGD/Cancer. Nevertheless, plasma-based classification could easily detect LGD samples (90% accuracy) while cyst fluid molecules’ concentrations were better for predicting HGD/Cancer samples (90% accuracy) (Table 2). The model had a low sensitivity in discriminating the three IPMN groups from each other when HGD and Cancer were considered separately. SCN samples form a distinct cluster from IPMN samples, whereas the other two groups overlap significantly in cyst fluid (Fig. 4A,B). The top 15 molecules in cyst fluid and plasma ranked by their VIP scores are presented in Fig. 4C,D. Interestingly, only a subset of metabolites, without lipids, were sufficient to achieve best performance. In particular, amino acids were the most important molecules for the classification of plasma samples.

**Table 2:** Performance measures of binary classifications with the chosen CPPLS-DA model.

		AUC^a^	Sensitivity	Specificity	Balanced accuracy^b^
SCN vs All	Cyst fluid	1.000	1.000	1.000	1.000
	Plasma	0.950	0.800	0.875	0.837
LGD vs All	Cyst fluid	0.935	0.875	0.913	0.894
	Plasma	0.825	1.000	0.812	0.906
HGD-Cancer vs All	Cyst fluid	0.949	0.889	0.923	0.906
	Plasma	0.854	0.636	1.000	0.818
IPMN vs SCN	Cyst fluid	1.000	1.000	1.000	1.000
	Plasma	1.000	1.000	1.000	1.000

^a^Area Under the ROC Curve; ^b^(Sensitivity + Specificity)/2.

### Correlations to clinical pancreatic blood markers

We next asked whether there was any significant correlation between the metabolites/lipids and the blood markers that are frequently used to define the IPMN patients. Pearson correlation analysis identified molecules in plasma showing strong correlations (r > 0.6 and adjusted p-value ≤ 0.05) with circulating CA19-9 and albumin, and to some extent with bilirubin (Supplementary Table S3). CA19-9 was found to be positively correlated with a total of 35 lipid molecules that were classified as CER (n = 1), FFA (n = 4), Phosphatidylcholines (PCs) (n = 10), Phosphatidylethanolamines (PE) (n = 10), Sphingomyelins (SM) (n = 4), and TAG (n = 5) (Supplementary Table S4a). In addition, albumin and bilirubin levels were positively correlated with 4-pyridoxate, adenine, carnitine, cysteine, and lipid molecules classified as CE (n = 1), FFA (n = 2), Lysophosphatidylcholines (LPC) (n = 2), Lysophosphatidylethanolamines (LPE) (n = 1), PE (n = 1), CER (n = 1), PC (n = 1), and TAG (n = 1) (Supplementary Table S4a). Negative correlations were noted between albumin and cystathionine, D-Ribose-5-P, inosinic acid or inosine monophosphate (IMP), and taurochenodeoxycholate and some lipids within the classes of PC (n = 9), TAG (n = 6) and CER (n = 6), while bilirubin negatively correlated with few metabolite and lipids (Supplementary Table S4b).

## Discussion

Pancreatic IPMNs are common precancerous lesions^6–8^. Today, only some radiological and clinical parameters are used to identify patients with high risk for cancer progression or malignancy, for example, main pancreatic duct dilatation, cyst diameter, rate of progression and elevated serum CA19-9^14–16^. Unfortunately, no high-accuracy tools are available that determine the IPMN-associated grade of dysplasia or that offer accurate differential diagnosis from other benign and low-risk PCNs (i.e. SCNs). These diagnostic limitations negatively affect patient management and treatment. Until recently, metabolites involved in IPMN disease progression have been scarcely studied^27^. A holistic view of the plasma and cyst fluid metabolic profile may aid the discovery of biomarkers capable of improving pre-operative PCN diagnosis.

We have shown that an integrated metabolomics and lipidomics approach can be used to 1) discriminate between IPMN and SCN and 2) determine the IPMN-associated grade of dysplasia. Our analysis offered superior predictive accuracy compared to conventional cross-sectional imaging or EUS-FNA^15^. The LOOCV balanced accuracy of discriminating Cancer/HGD from SCN and LGD was 90.6% for cyst fluid and 81.8% for plasma. When discriminating IPMN as a whole from SCN, accuracy reached 100% for both plasma and cyst fluid. The availability of accurate plasma-based tests could represent a major advantage for patients who do not require invasive procedures like EUS-FNA, which are associated with risk of complications and low-diagnostic accuracy^31^.

While previous PC metabolome studies measured around 50-100 metabolites per case^24,26,29,32^, our high-definition integrated approach measured around 100 metabolites from 15 different biological classes and 1000 lipid molecular species from 13 different lipid classes, largely covering the important metabolome spectrum, i.e. sugars, nucleotides, amino acids, and lipids. Recent elegant metabolome studies^24,28^ pointed out that a number of compounds, including very long-chain fatty acids, phospholipids, and taurine, were differentially present in PC patients or PC tissue compared to healthy subjects or parenchyma tissue, respectively. This agrees with our findings of enriched taurine and fatty acid metabolism pathways and phospholipid biosynthesis pathways in cyst fluid of pre-malignant or early malignant cases, e.g. and LGD and Cancer/HGD, as compared to SCN. Moreover, our study also has shown significant alterations of different classes of molecules, mainly TAGs, detected in plasma of Cancer/HGD, and LGD, compared to SCN. We did not observe significant alterations of molecules in plasma when comparing HGD/Cancer with LGD, suggesting these disease groups display a more comparable plasma profile.

The tumor marker CA19-9 is used for predicting malignancy of IPMN and monitoring PC progression, but its use as a definitive diagnostic marker, especially detecting IPMN HGD, is limited^33,34^. Combining additional blood markers with CA19-9 was recently shown to improve early detection of PC^33^, and building a broader molecular profile around CA19-9 in IPMN patients may enhance the diagnostic accuracy. The lipid metabolites strongly associated with CA19-9 in this cohort are therefore of interest and need to be examined further, possibly together with metabolites correlating with serum albumin and bilirubin. Our findings that plasma, but not cyst fluid metabolites, strongly correlated with these three markers, suggest that systemic, rather than local factors, may have an influence on development and progression of IPMN.

A strength of this paper is the investigation of a surgical cohort of patients, with definitive histology and assessment of the grade of dysplasia. This allowed us to accurately match the actual IPMN-associated grade of dysplasia with our data, avoiding problems of misdiagnosis that occur in more than one third of the patients undergoing EUS-FNA with cyst fluid analysis^12^. In addition, we used a validated targeted and (semi) quantitative analysis through a robust and reliable LC-MS/MS approach with strict quality management^35^. We furthermore tested several linear mixed models to assess many covariate parameters (see Table 1), and while use of statins was considered as possible confounding factor, it did not improve the final classification model which only adjusted for age and BMI.

However, this study has also some limitations. Firstly, there is possible selection bias because we analyzed a small and homogenous cohort of patients which might not be fully representative of the entire population and thus might potentially restrict the predictive power of lipidomic/metabolomics profiling to certain groups within the general population. In this relatively small cohort all SCNs cases are female, which is not surprising as the prevalence rate in the general population for SCNs is 9-16% of all cystic lesions while approximately 75% of patients with SCNs are women^36^. Although this gender imbalance can be certainly considered a limitation we observed that, after adjusting for BMI and age, the effect of sex did not have any impact on out-of-sample model prediction accuracy. Therefore, we believe that our classification accuracy is mainly a consequence of differences between diseases while we expect unbalances in sex distribution to have only a very negligible influence. Moreover, we did not include other types of mucinous tumors of the pancreas, such as mucinous cystic neoplasm (MCN). However, such lesions are easier to recognize, due the peculiar epidemiological and radiological features (typically in body and tail of the pancreas, almost exclusively in young females) that make diagnosis not particularly challenging^37^. Additionally, the current study might suffer from a possible sampling bias, considering that fluid was aspirated from one or two accessible cysts, despite IPMNs often being multifocal and occurring in different locations of the pancreas (head/body/tail). Therefore, one cannot exclude the possibility that metabolic profiles of the sampled cysts might not be representative of all cystic lesions. Lastly, we did not investigate possible factors that might have potential effects on (lipid) metabolism, such as specific genetic mutations (e.g GNAS or KRAS gene)^38,39^.

This study has comprehensively mapped the metabolite and lipid makeup of cyst fluid and plasma from PCN patients with defined pathology, using integrated metabolomics and lipidomics. Our findings have clinical implications and may support assay development for differential diagnostics of PCNs to improve patient management. Future studies are needed to test larger patient cohorts using the proposed model, to better understand associations between metabotypes and IPMN malignancy risks.

## Methods

### Study population and ethical considerations

In this prospective cohort study, patients undergoing pancreatic surgery for suspected pancreatic cystic neoplasm (PCN) with post-surgically validated intraductal papillary mucinous neoplasm (IPMN) and serous cystic neoplasm (SCN) from February 2016 to January 2017 at Karolinska University Hospital, Sweden, were included. Excluded were cases without a cystic component, non-IPMNs, or those without cyst fluid in the resected pancreas (Supplementary Fig. S1). This study follows the Helsinki convention and good clinical practice with permission of the Ethical Review Board Stockholm and the Karolinska Biobank Board (Dnr 2015/1580-31/1). Written informed consent was obtained from all patients.

### Pancreatic cyst fluid collection

Fresh resection specimens were received at the pathology laboratory within 20 minutes of surgical removal, in sterile conditions and on ice. Macroscopic assessment to identify the cystic lesion and main pancreatic duct was done by a specialist pancreatic pathologist. Fluid from the main pancreatic duct was collected using a syringe without needle. When the cystic lesion was readily identified in the intact specimen, the fluid was aspirated using a syringe with needle. For specimens in which the cystic lesion was not readily accessible from the surface the specimen was cut or when the cyst content was too viscous content was aspirated using a syringe without needle. Aspirated fluid was stored at −80 °C until further analysis.

### Peripheral blood collection and plasma isolation

Venous whole blood was collected in K_2_ EDTA Vacutainer® tubes (BD, Stockholm, Sweden) immediately prior to surgery. Within four hours of collection, blood was processed using Ficoll Paque Plus (GE Life Sciences, Uppsala, Sweden) gradient-density centrifugation following manufacturer’s instructions and the plasma fraction was stored at −80 °C until further analyses.

### Histopathological diagnosis and cyst fluid classification

Resection specimens were fixed in 4% formaldehyde and processed for routine histopathological diagnosis. The cystic lesions were classified by light microscopic examination of hematoxylin-eosin stained slides by a specialized pancreatic pathologist as IPMN or SCN^40^. The grade of dysplasia in IPMN was assessed using a 2-grade (high/low) scale, according to current international standard^41^. To make the cyst fluid classification more representative of the neoplastic epithelium that produces it, specimens showing <5% high-grade dysplasia (HGD) were classified as low-grade dysplasia (LGD). Specimens with concomitant invasive carcinoma were classified as “Cancer” and considered as a separate class for further analyses.

### Chemicals

All metabolite standards used in the analysis were purchased from Sigma-Aldrich (Helsinki, Finland), while isotopically labelled metabolite internal standards (IS) were obtained from Cambridge Isotope Laboratory (Tewksbury, MA, USA). For lipidomics, kits containing 50 labelled internal standards across 13 lipids classes were purchased from SCIEX (Framingham, MA, USA). Ammonium formate, ammonium acetate, and ammonium hydroxide were obtained from Sigma-Aldrich (Helsinki, Finland). Formic acid (FA), acetonitrile (ACN), methanol (HiPerSolv CHROMANORM, LCMS grade), ethyl acetate (HPLC grade), 2-propanol, 1-propanol, and dichloromethane were purchased from VWR International (Helsinki, Finland). Deionized water, up to a resistivity of 18 MΩ⋅cm, was purified with a Barnstead Easypure RoDi water purification system (Thermo Scientific, Marietta, OH, USA). Whole blood was purchased from the Finnish Red Cross blood service (Helsinki, Finland) from which serum samples were prepared and used as internal quality control samples.

### Metabolomic analysis

Metabolomic analysis of samples was performed using liquid chromatography-mass spectrometry as previously described in the supplementary data of Nandania *et al*.^35^. Briefly, 15 labeled internal standards were used to estimate quantitative levels of 100 metabolites. To 100 µL thawed sample (plasma or cyst fluid), 10 µL of labelled internal standard mixture was added, and then metabolites were extracted using protein precipitation by adding acetonitrile +1% formic acid (1:4, sample:solvent). The collected extracts were dispensed in Ostro 96-well plates (Waters Corporation, Milford, USA) and filtered by applying a vacuum at a delta pressure of 300–400 mbar for 2.5 min on robot’s vacuum station. Filtered sample extract (5 µL) was injected in an Acquity UPLC-system coupled to a Xevo TQ-S triple quadrupole mass spectrometer (Waters Corporation, Milford, MA, USA) which was operated in both positive and negative polarities with switching time of 20 milliseconds. Multiple Reaction Monitoring (MRM) acquisition mode was selected for the quantification of metabolites. MassLynx 4.1 software was used for data acquisition, data handling and instrument control. Data processing was done using TargetLynx 4.1 software.

### Lipidomic analysis

Lipids were extracted with liquid-liquid extraction (LLE) method using ethyl acetate and methanol. In borosilicate glass tubes, to 100 µL thawed sample (plasma or cyst fluid), 1 mL methanol and 1 mL water was added. Then, 100 μL of labelled internal standard mixture (prepared as per SCIEX LIPIDYZER manual’s instructions) was added and allowed to equilibrate with the samples. To each tube 3.5 mL of ethyl acetate was added after which tubes were put on a rotator shaker for 15 min at 30 RPM, followed by centrifugation at 3000 RPM for 10 min. After centrifugation, the upper layer of ethyl acetate was collected and dried under N_2_ gas. Dried samples were reconstituted with 250 μL of mobile phase (dichloromethane:methanol (50:50) containing 10 mM ammonium acetate) for injection. Lipid separation and quantitation was performed on the SCIEX Lipidyzer^™^ platform using a SCIEX 5500 QTRAP® mass spectrometer (SCIEX, Washington, D.C., USA) with SelexION® Differential ion mobility (DMS) technology by directly infusing 50 μL of extracted samples with a mobile phase at flow rate of 70 µL/min. Two acquisition methods, with and without SelexION® technology, were used to cover 13 lipid classes using flow injection analysis. The lipid molecular species were measured using MRM strategy in both positive and negative polarities. Positive ion mode was used for the detection of lipid classes – sphingomyelins (SM), diacylglycerols (DAG), cholesteryl esters (CE), ceramides (CER), triacylglycerols (TAG), and negative ion mode was used for the detection of lipid classes – lysophosphatidylethanolamines (LPE), lysophosphatidylcholines (LPC), phosphatidylcholines (PC), phosphatidylethanolamines (PE) and free fatty acids (FFA). Lipidomics Workflow Manager software was used for acquisition of samples, automated data-processing, signal detection and lipid species concentration calculations.

### Statistical analysis

All data analyses were performed with R 3.5.1 and Stan 2.17.1^42,43^. Concentration values in μmol/L were assumed to follow a lognormal distribution and were therefore log-transformed as a preliminary normalization operation. Missing values were removed from the dataset following the “modified 80% rule”, according to which a variable is discarded if the relative frequency of missing values is more than 0.8 in all clinical groups^44^. Remaining missing values were imputed with the QRILC function from R package imputeLCMD^45^. Considering the lack of balance for basic clinical parameters (Table 1), all values were adjusted for the effects of confounding factors using a linear mixed model. Let ${y}_{gij}$ be the log-concentration of molecule *j* observed at sample *i* belonging to phenotypic group *g*. The basic assumption of our model is that observations are conditionally independent and normally distributed, with same standard deviation σ but different mean value μ:

$${y}_{gij}|{\mu }_{gij},\,\sigma  \sim N({\mu }_{gij},\,{\sigma }^{2})$$

Covariates to include in the model were selected by the highest out-of-sample point-wise predictive accuracy^46^. Parameter μ was then defined as the linear combination

$${\mu }_{gij}=\theta +{\varphi }_{i}+{\lambda }_{j}+{\eta }_{gj}+{\gamma }_{1j}ag{e}_{i}+{\gamma }_{2j}bm{i}_{i}$$

where θ is the grand mean, ${\varphi }_{i}$ is the general effect of sample *i*, ${\lambda }_{j}$ is the effect of molecule *j*, ${\eta }_{gj}$ is the effect of phenotypic group *g* on molecule *j*, and ${\gamma }_{v}(v=1,2)$ are effects varying with molecules. Fold change of molecule *j* between groups *g* and *h* was therefore defined as ${e}^{({\eta }_{gj}-{\eta }_{hj})}$. All coefficients associated with the same discrete category are constrained to sum to zero in order to make the model identifiable. We assigned to each unknown parameter weakly informative prior distributions as follows:

$$\theta  \sim t(3,\,0,\,10)$$

$${\varphi }_{i} \sim N(0,\,{\alpha }^{2}),\,i=1,\ldots ,n$$

$${\lambda }_{j} \sim N(0,\,{\beta }^{2}),\,j=1,\ldots ,\,m$$

$${\gamma }_{vj} \sim N(0,{\omega }_{v}^{2}),\,v=1,\,2,\,j=1,\ldots ,\,m$$

$${\eta }_{gj} \sim N(0,{\zeta }_{g}^{2}),g=1,\ldots ,\,k,\,j=1,\ldots ,\,m$$

$$\sigma  \sim halft(3,0,10)$$

$$\alpha  \sim halft(3,0,10)$$

$$\beta  \sim halft(3,0,10)$$

$${\omega }_{v} \sim halft(3,0,10),\,v=1,\,2$$

$${\zeta }_{g} \sim halft(3,0,10),\,g=1,\ldots ,\,k$$

where *t* refers to the three-parameters Student’s *t*-distribution and *halft* to the same distribution but truncated at 0 and defined only on the positive values^47^. Total number of phenotypic groups *k* depended on the particular statistical analysis being conducted. Estimated fold changes and their corresponding 95% credibility intervals, computed from 20,000 posterior samples, are available in Supplementary Table S1. Prior to visualization, classification, and enrichment analyses, the dataset was adjusted for the confounding covariates “age” and “BMI” and subsequently standardized to a mean of zero and unit variance. Principal Component Analysis (PCA) was applied to the adjusted data for exploratory data purposes. Heatmaps and data projection on the first two principal components were used to visualize the dataset. Classification was performed using a Canonical Powered Partial Least Squares Discriminant Analysis (CPPLS-DA), fitted with the *pls* R package^48,49^. Classification performance was measured with a Leave-One-Out Cross Validation (LOO-CV) strategy, and balanced accuracy (average between sensitivity and specificity of the classifier) is reported^50^. Best explanatory molecules were selected according to their Variable Importance in Projection (VIP) ranking scores according to the following iteration scheme^51^. At each step of the algorithm the performance of the model was recorded with a LOO-CV strategy and the molecules were sorted according to their VIP score. Subsequently, 5% of the molecules with the lowest VIP score were discarded and this operation was repeated until the number of molecules allowed model identifiability. The model with the highest performance was ultimately selected. Pathway enrichment analysis (QEA) was performed with the free web service MetaboAnalyst 4.0^52^. All figures were generated in R 3.5.1^42^.

### Ethical considerations

This study follows the Helsinki convention and good clinical practice. This study was conducted at Karolinska University Hospital under permission of the Ethical Review Board Stockholm and the Karolinska Biobank Board (Dnr 2015/1580-31/1). Written informed consent was obtained from all patients.

## Supplementary information

Supplementary Material

Supplementary Table S1

Supplementary Table S2

Supplementary Table S3

## Data Availability

The raw datasets generated during the current study are available from the corresponding author on reasonable request. Results from analyzed datasets are available in Supplemental Tables 1–3.
