# Author Correction: Integrated targeted metabolomic and lipidomic analysis: A novel approach to classifying early cystic precursors to invasive pancreatic cancer

**DOI:** 10.1038/s41598-022-08527-z

**Published:** 2022-03-28

**Authors:** Rogier Aäron Gaiser, Alberto Pessia, Zeeshan Ateeb, Haleh Davanian, Carlos Fernández Moro, Hassan Alkharaan, Katie Healy, Sam Ghazi, Urban Arnelo, Roberto Valente, Vidya Velagapudi, Margaret Sällberg Chen, Marco Del Chiaro

**Affiliations:** 1 Division of Clinical Diagnostics and Surgery, DENTMED, Karolinska Institutet, Huddinge, Sweden; 2 Metabolomics Unit, Institute for Molecular Medicine Finland (FIMM), HiLIFE, University of Helsinki, Helsinki, Finland; 3 Division of Surgery, CLINTEC, Karolinska University Hospital, Stockholm, Sweden; 4 Division of Pathology, Department of Clinical Pathology/Cytology, Karolinska University Hospital, Huddinge, Sweden; 5 Division of Pathology, LABMED, Karolinska Institutet, Huddinge, Sweden; 6 Department for Digestive Diseases, Sapienza University of Rome, Rome, Italy; 7 Division of Surgical Oncology, Department of Surgery, University of Colorado Denver, Aurora, CO USA; 8 Tenth People’s Hospital, Tongji University, Shanghai, China; 9 College of Dentistry, Prince Sattam bin Abdulaziz University, Al-Kharj, Saudi Arabia

Correction to: *Scientific Reports* 10.1038/s41598-019-46634-6, published online 15 July 2019

The original version of this Article omitted an affiliation for Hassan Alkharaan. The correct affiliations are listed below.

Division of Clinical Diagnostics and Surgery, DENTMED, Karolinska Institutet, Huddinge, Sweden.

College of Dentistry, Prince Sattam bin Abdulaziz University, Al-Kharj, Saudi Arabia

The original Article has been corrected.

