# Supplementary material for: Integrated targeted metabolomic and lipidomic analysis: A novel approach to classifying early cystic precursors to invasive pancreatic cancer
Source: Sci Rep. 2019 Jul 15;9:10208. doi: 10.1038/s41598-019-46634-6 (PMC6629680; doi:10.1038/s41598-019-46634-6)
Supplement: Supplementary file 1 — Supplementary Material [file 41598_2019_46634_MOESM1_ESM.pdf]

Integrated targeted metabolomic and lipidomic analysis: A novel approach to classifying early cystic precursors to invasive pancreatic cancer

Rogier Aäron Gaiser, Alberto Pessia, Zeeshan Ateeb, Haleh Davanian, Carlos Fernández Moro, Hassan Alkharaan, Katie Healy, Sam Ghazi, Urban Arnelo, Roberto Valente, Vidya Velagapudi, Margaret Sällberg Chen, Marco Del Chiaro

Supplementary Figures and Tables

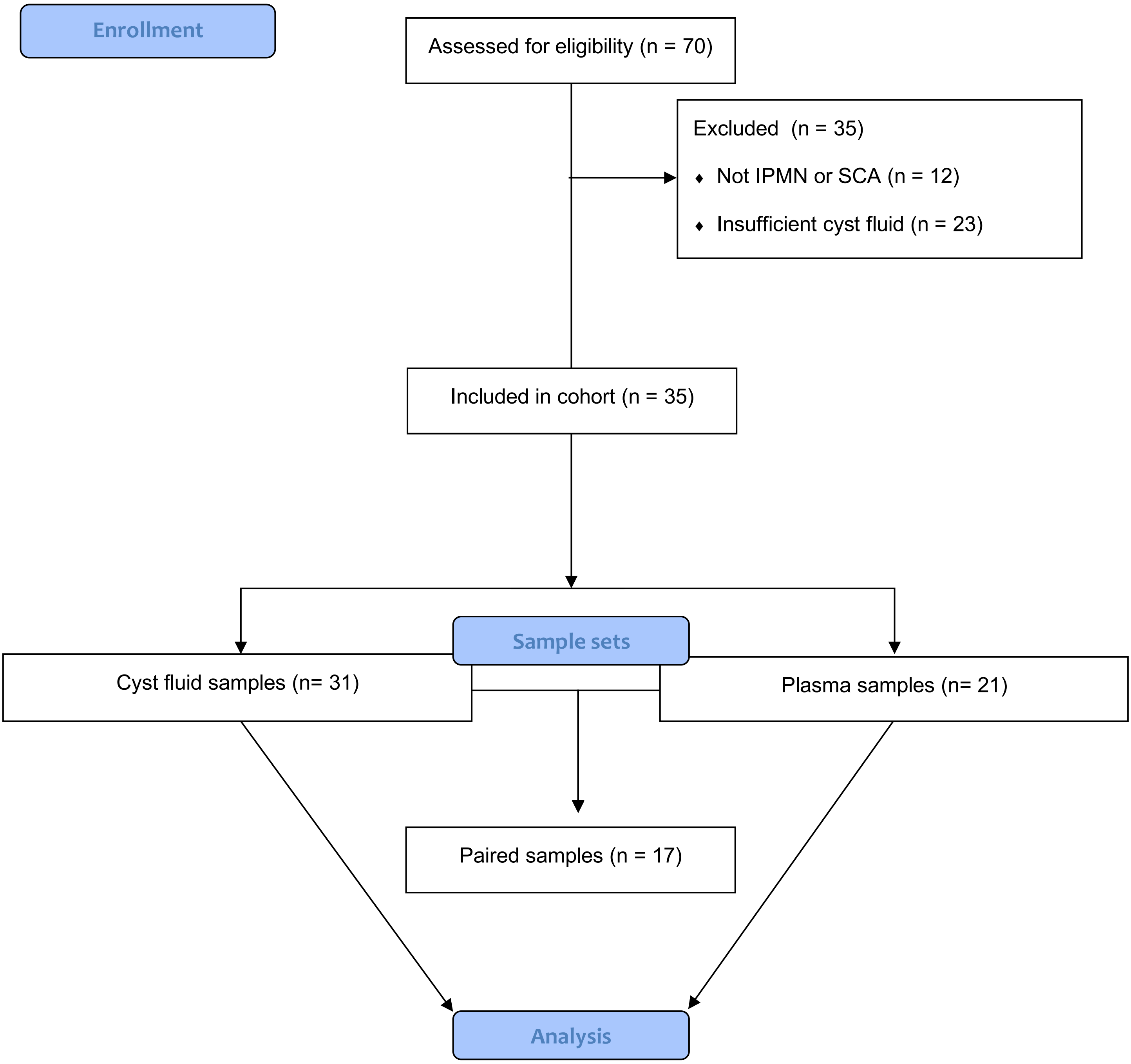

Supplementary Figure S1. Flow chart depicting the study design and number of patients from whom cyst fluid and/or plasma was analyzed.

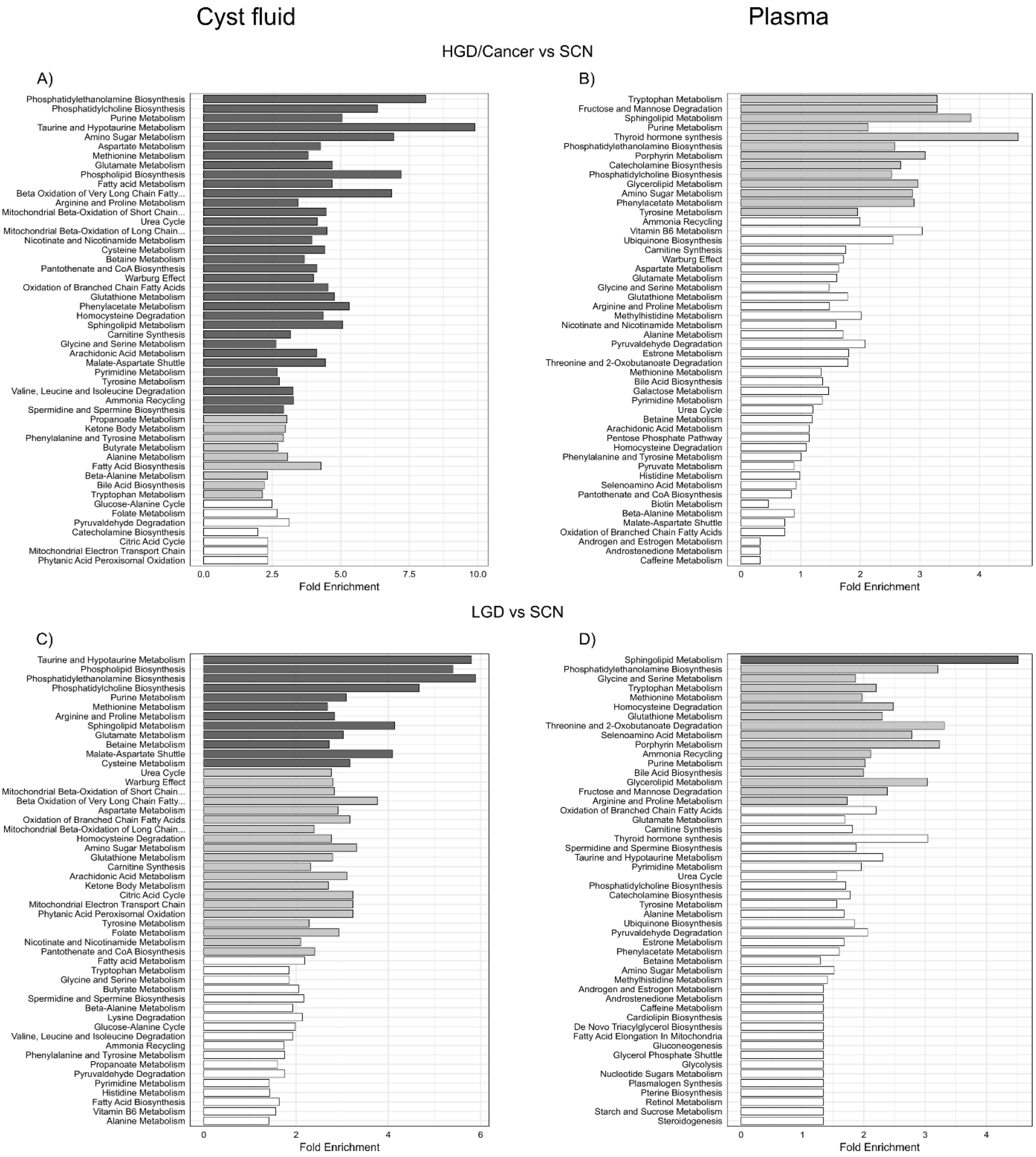

Supplementary Figure S2. Quantitative enrichment analysis of metabolic pathways. Serous cystic neoplasm (SCN) group comparisons against high-grade dysplasia (HGD) and Cancer patients in cyst fluid **(A)** and plasma **(B)** and against low-grade dysplasia (LGD) patients in cyst fluid **(C)** and plasma **(D)**. Dark grey colour represents pathways with an adjusted p-value < 0.05. Light grey colour represents pathways with an unadjusted p-value < 0.05.

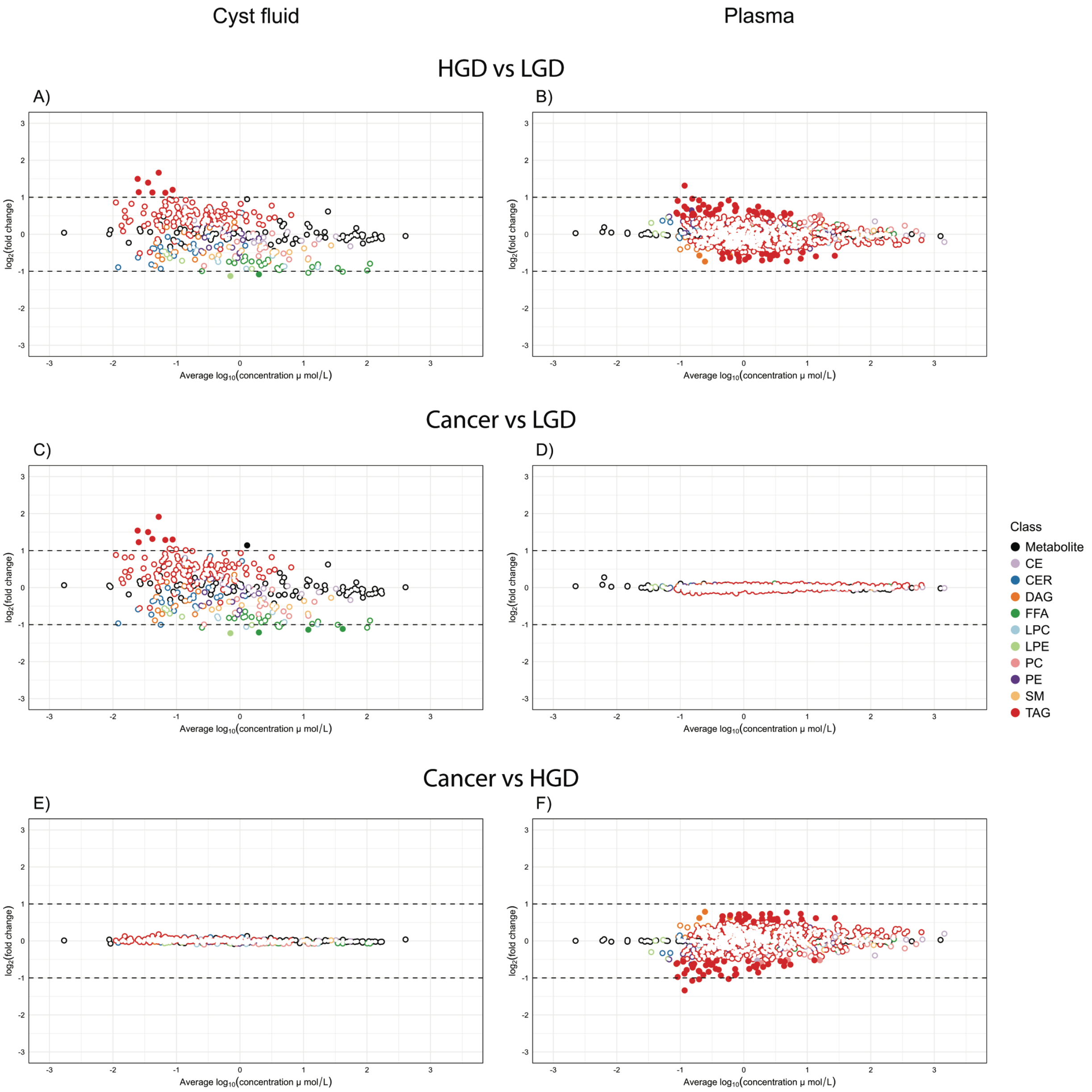

Supplementary Figure S3. Estimated fold changes of concentrations of all measured analytes (including metabolite and lipid molecular species) between selected groups. Concentrations of HGD compared to LGD in cyst fluid **(A)** and plasma **(B)**. Concentrations of Cancer compared LGD in cyst fluid **(C)** and plasma **(D)**. Concentrations of Cancer compared HGD in cyst fluid **(E)** and plasma **(F)**. Filled dots are fold changes whose credibility interval does not overlap with the null reference value of one-fold change, or zero on the plotted log scale.

Supplementary Table S4a. Positive correlations associated with clinical blood markers.

Blood marker	Type	Class	Name
CA19-9	Lipid	Ceramides	CER(22:0)
		Free fatty acids	FFA(12:0);FFA(14:0); FFA(14:1);FFA(18:0)
		Phosphatidylcholines	PC(18:2/20:2);PC(16:0/18:2);PC(16:0/20:1); PC(16:0/18:3); PC(18:2/18:2); PC(18:1/18:2); PC(12:0/18:1); PC(18:1/18:3); PC(16:0/12:0); PC(14:0/18:2)
		Phosphatidylethanolamines	PE(18:2/16:1); PE(18:0/18:0); PE(O-18:0/18:2); PE(O-18:0/18:1); PE(18:1/18:2); PE(18:1/22:6); PE(P-16:0/18:2); PE(18:2/18:2); PE(P-18:0/18:2); PE(18:0/18:2)
		Sphingomyelins	SM(20:1); SM(20:0); SM(14:0); SM(22:0);
		Triacylglycerols	TAG51:4-FA18:2; TAG52:6-FA18:2; TAG53:4-FA18:2; TAG55:1-FA18:1; TAG53:4-FA17:0; TAG55:1-FA18:1; TAG53:4-FA17:0
Albumin	Metabolite	Pyridines and derivatives	4-Pyridoxate
		Purines and derivatives	Adenine
		Quaternary ammonium salts	Carnitine
		Alpha amino acids	Cysteine
	Lipid	Cholesteryl esters	CE(20:5)
		Free fatty acids	FFA(18:4); FFA(24:0)
		Lysophosphatidylcholines	LPC(18:0); LPC(22:6)
		Lysophosphatidylethanolamines	LPE(22:6)
		Phosphatidylethanolamines	PE(O-16:0/20:4); PE(O-16:0/22:6); PE(O-18:0/22:5); PE(P-18:0/22:5); PE(P-18:0/22:6)
Bilirubin	Metabolite	Purines and derivatives	IMP
	Lipid	Ceramides	CER(14:0)
		Phosphatidylcholines	PC(16:0/16:0)
		Triacylglycerols	TAG36:0-FA12:0

Correlation cut-off at 0.6. Adjusted p-value < 0.05.

Supplementary Table S4b. Negative correlations associated with clinical blood markers.

Blood marker	Type	Class	Name
Albumin	Metabolite	Alpha amino acids and derivatives	Cystathionine
		Carbohydrates and carbohydrate conjugates	D-Ribose-5-P
		Purines and derivatives	IMP
		Bile acids, alcohols and derivatives	Taurochenodesoxycholate
	Lipid	Ceramides	CER(14:0); CER(16:0); CER(18:0); CER(20:0); CER(22:1); CER(24:1)
		Lactosylceramides	LCER(16:0)
		Phosphatidylcholines	PC(15:0/18:1); PC(15:0/18:2); PC(16:0/16:0); PC(16:0/18:0); PC(16:0/18:1); PC(17:0/18:1); PC(17:0/18:2); PC(18:1/18:1); PC(20:0/18:1)
		Phosphatidylethanolamines	PE(18:0/18:1)
		Triacylglycerols	TAG36:0-FA12:0; TAG53:4-FA20:4; TAG53:5-FA20:4; TAG55:5-FA20:4; TAG58:6-FA20:4; TAG58:8-FA20:4
Bilirubin	Metabolite	Alpha amino acids	Cysteine
		Pyrimidines and derivatives	Orotate
		Quaternary ammonium salts	Carnitine
	Lipid	Cholesteryl esters	CE(18:2);CE(20:5)
		Phosphatidylethanolamines	PE(P-18:0/22:5)

Correlation cut-off at -0.6. Adjusted p-value < 0.05.
